# Dr. Frank W. Maloney

**Published:** 1915-11

**Authors:** 


**Dr. Frank W. Maloney,** Buffalo 1891, died at his home in Rochester, Aug. 23, aged 51.

